# Primary Human mDC1, mDC2, and pDC Dendritic Cells Are Differentially Infected and Activated by Respiratory Syncytial Virus

**DOI:** 10.1371/journal.pone.0016458

**Published:** 2011-01-28

**Authors:** Teresa R. Johnson, Christina N. Johnson, Kizzmekia S. Corbett, Gretchen C. Edwards, Barney S. Graham

**Affiliations:** Viral Pathogenesis Laboratory, Vaccine Research Center, National Institute of Allergy and Infectious Diseases, National Institutes of Health Bethesda, Bethesda, Maryland, United States of America; University of Georgia, United States of America

## Abstract

Respiratory syncytial virus (RSV) causes recurrent infections throughout life. Vaccine development may depend upon understanding the molecular basis for induction of ineffective immunity. Because dendritic cells (DCs) are critically involved in early responses to infection, their interaction with RSV may determine the immunological outcome of RSV infection. Therefore, we investigated the ability of RSV to infect and activate primary mDCs and pDCs using recombinant RSV expressing green fluorescent protein (GFP). At a multiplicity of infection of 5, initial studies demonstrated ∼6.8% of mDC1 and ∼0.9% pDCs were infected. We extended these studies to include CD1c^−^CD141^+^ mDC2, finding mDC2 infected at similar frequencies as mDC1. Both infected and uninfected cells upregulated phenotypic markers of maturation. Divalent cations were required for infection and maturation, but maturation did not require viral replication. There is evidence that attachment and entry/replication processes exert distinct effects on DC activation. Cell-specific patterns of RSV-induced maturation and cytokine production were detected in mDC1, mDC2, and pDC. We also demonstrate for the first time that RSV induces significant TIMP-2 production in all DC subsets. Defining the influence of RSV on the function of selected DC subsets may improve the likelihood of achieving protective vaccine-induced immunity.

## Introduction

Respiratory syncytial virus (RSV) is a pneumovirus in the family Paramyxoviridae and has a non-segmented negative-sense single-stranded RNA genome [1]. RSV is a major cause of respiratory disease in infants, the elderly, and recipients of bone marrow or lung transplants. Infants who experience severe disease are at significant risk for development of wheezing and hyperreactive airways disease in later childhood [2]–[4]. More than 95% of children are infected with RSV by 2 years of age, and ∼50% of children infected in the first year of life are reinfected during the second year [5]. Therefore, natural RSV infection fails to induce immunity that prevents reinfection despite relatively minor genetic variation between strains [6], [7]. Inefficient function in memory T and B cell compartments has been described [8]–[11], and adults are reinfected throughout life. Approximately 25% of healthy adults repeatedly challenged intranasally with the identical strain of RSV could be reinfected [7]. Reinfection during childhood causes significant morbidity [12], and in adults with normal immune function, symptoms are typically restricted to the upper airway. Antibody responses induced by natural infection in infants have been reported to be of relatively low magnitude and short-lived [13], but when the titers of pre-existing maternal antibody are low, infection-induced antibodies are generally sufficient to protect the lower respiratory tract [14]. RSV is also known to suppress the proliferative capacity of lymphocytes [15]. Thus, there is evidence that RSV interferes with development of both humoral and cell-mediated immune mechanisms which results in an overall immunological state that cannot protect the upper airway from reinfection.

Two distinctive features of the immune response to RSV, allergic Th2-biased inflammation associated with severe neonatal disease and failure to induce protective immunity, suggest induction of inappropriate and ineffective RSV-specific immune responses occurs at the time of initial antigen exposure. Dendritic cells (DCs) may represent the earliest encounter between the virus and the host immune system. Distributed at mucosal surfaces (i.e. portals of entry) and throughout the body in organs and blood, DCs serve as professional antigen-presenting cells (APCs). DCs are organized into phenotypic and functional subsets [16]. The two major classes are CD11c^+^ myeloid DCs (mDCs) and CD11c^−^ plasmacytoid DCs (pDCs) with distinct and complementary roles in the induction of immune responses. Additional distinctions can be made within the mDC subset with recent identification of CD1c^−^CD141^+^ DCs, termed mDC2, while the prototypic mDC, now designated mDC1, are CD1c^+^CD141^−^ [17]–[20]. mDCs are efficient in uptake, processing, and presentation of foreign antigens while pDCs are less effective in these processes. Upon encounter with antigen, both mDCs and pDCs undergo maturation, upregulating CD80, CD83, CD86, CD40, and major histocompatibility class II, and become more efficient at T and B cell activation. Upon contact with antigen, DCs are activated to secrete an array of cytokines and chemokines. This is of particular importance to antiviral immunity as pDCs can be triggered to produce high levels of type I interferons upon exposure to virus [21]–[25]. Secretion of IFN-α has been suggested as an important factor to subsequent maturation of mDCs [21], [22], [24]. This division of labor may be of special significance at mucosal sites such as the lung as recently reviewed [26]. RSV has evolved multiple mechanisms to interfere with induction and effector functions of type I interferons [1]. The nonstructural (NS) proteins 1 and 2 are first in the gene order, signifying their importance, and appear to be entirely devoted to the inhibition of IFN pathways [27].

Monocytes isolated from neonates or from adults exhibit markedly different cytokine profiles following exposure to RSV with monocytes from adults producing high levels of cytokines associated with innate and adaptive immunity [28]. In contrast, neonatal monocytes produced a more limited set of cytokines and did not proliferate following RSV exposure, suggesting suppression of adaptive immunity in neonates may contribute to RSV-induced disease in early life. Similarly, RSV has been shown to infect cord blood-derived DCs, increasing surface expression of maturation markers and cytokine production [29], [30]. Furthermore, RSV-infected cord-blood DCs co-cultured with autologous T cells induced T cell proliferation, but suppressed T cell-derived IFN-γ production [30]–[32].

Most studies examining RSV interactions with human DCs have been conducted using monocyte-derived DCs (moDCs) generated *in vitro* from adult peripheral blood monocytes. As with cord-blood derived DCs, RSV was shown to infect and mature moDCs [33]–[36]. When co-cultured with autologous T cells, RSV-exposed moDCs suppressed cytokine production and T cell proliferation [33], [34], [36]–[38]. The age of the DC donor was shown to influence the magnitude of T cell suppression [38]. Type 2 cytokine production could be suppressed without concomitant induction of type 1 cytokine production [33]. Chi et. al. demonstrated IFN-α and IFN-λ in supernatants from moDCs suppressed proliferation of CD4 T cells [37]. When exposed to recombinant RSV having deletions of NS1, alone or in conjunction with NS2, the percentage of RSV-infected moDCs were reduced, but cytokine production and expression of selected maturation markers were increased [39]. Antibody blocking of the type I IFN receptor increased the number of RSV-infected moDCs, but inhibited maturation, leading the authors to suggest that NS1 and NS2 proteins suppress moDC maturation by antagonism of type 1 IFNs. In addition to maturation markers, RSV infection selectively increased expression of ligands for NK cell receptors, demonstrating the potential for RSV to regulate innate immunity [40].

Primary DCs have also been studied in human, bovine, and ovine RSV infection. RSV infection has been shown to induce infiltration of mDCs and pDCs and cytokine production in the respiratory tract of children during acute infection with concomitant decreases of both cell types in the blood [41], [42]. Influenza also increased DC recruitment and cytokine levels in this study, but greater numbers of mDC1 and pDC2 in the respiratory tract and higher levels of MCP-1 in the nasal wash and RANTES in the serum in influenza-infected infants suggested that RSV and influenza induce distinct patterns of innate immune responses [41]. DC numbers in the blood of children at age 6 yrs who were hospitalized with bronchiolitis during primary RSV infection demonstrated that those children diagnosed with asthma by age 6 had fewer mDCs and pDCs than did non-asthmatic study participants with greater deficits found in pDC numbers [43]. A limited number of e*x vivo* studies examining the effects of RSV exposure on primary pDCs have been performed [44], [45], demonstrating that RSV Long induces IFN-α production [44] while RSV A2 appears to inhibit IFN-α production mediated by TLR7 [45]. Similarly, preexposure of pDCs to RSV reduced production of IFN-α elicited by TLR3 or TLR9 agonists [34].

While present at a much lower frequency than mDC1 and pDC in the circulation [19], [20], mDC2 are found in the lung at a 2–3-fold greater frequency than mDC1 and 3–6-fold greater frequency than pDC [46], [47]. The identification of numerous phenotypic and functional differences among pulmonary mDC2 and mDC1 or pDC suggests a possible preferential role for mDC2 in generating or regulating immunity and disease pathogenesis in the respiratory tract distinct from that of mDC1 and pDC [46], [48]. The case for distinct roles in host immunity for each human DC subset is further strengthened by recent publications from four independent labs showing human CD141+ mDC2 are the functional equivalent of mouse CD8α+ DCs, capable of cross-presentation of exogenous antigens [49]–[53]. Furthermore, the increased frequency of mDC2 in the circulation of children with acute asthma [54] makes interactions between RSV and mDC2 of particular interest. Here we examine the effects of exposure to RSV on primary mDCs and pDCs, in parallel with moDCs and macrophages derived *in vitro* from monocytes from the same donor, using recombinant RSV A2 expressing GFP (rgRSV). Incorporating rgRSV into a multiparameter flow cytometry panel allows simultaneous analyses of infection rate, viability, and 4–8 additional cellular factors, such as maturation markers, on a single cell basis. Using this experimental system, we evaluated the impact of RSV exposure on the infection and maturation of DCs and demonstrate that mDC1 and mDC2 are infected at similar levels, and at a rate about 5-fold higher than pDC. In addition, we describe the patterns of maturation and cytokine production in mDC1, mDC2, and pDC compared to moDCs and macrophages. This is the first detailed analyses of the impact of RSV exposure on primary human myeloid dendritic cells and provides a benchmark for future studies on the impact of RSV on dendritic cell function.

## Materials and Methods

### Viruses

Recombinant RSV expressing GFP (rgRSV), a gift of Mark Peeples (Ohio State University) [55] was grown in HEp-2 cells with MEM containing 10% FCS. To remove cytokines produced by HEp-2 cells (purchased from the American Type Culture Collection) during growth of the virus stock, sucrose-purified rgRSV stocks were. rgRSV was UV-inactivated by exposing virus to 75,000 mJ/cm2 of ultraviolet light using a StrataLinker (Stratagene). As a mock-infection control, uninfected HEp-2 cells were processed in the same manner as done for RSV stocks.

### Isolation of primary DCs and generation of moDCs and macrophages

Elutriated human monocytes were obtained from the NIH Department of Transfusion Medicine. Blood samples were obtained from paid healthy volunteers who gave written informed consent to participate in a study for the collection of blood samples for in vitro research use approved by the National Cancer Institute Central Institutional Review Board. The protocol is designed to protect subjects from research risks as defined in 45CFR46 and to abide by all NIH guidelines for human subjects research (protocol number 99-CC-0168). The donors, who remained anonymous to the investigators, were healthy adults of known age, gender, race, and serologic status for HIV-1 and cytomegalovirus (CMV). pDCs, mDC1, and mDC2 were sequentially purified using BDCA-4, BDCA-1, then BDCA-3 magnetic isolation kits (Miltenyi Biotec). Isolated pDCs were incubated in 10% RPMI and IL-3 (1 ng/ml, BioWhittaker). Isolated mDC1 and mDC2 were cultured in 10% RPMI and GM-CSF (2 ng/ml, PeproTech Inc.). Purity of the isolated DCs was examined by phenotypic analyses using a lineage cocktail (CD3, CD14, CD16, CD19, CD20, CD56), and CD1c, CD11c, CD14, CD123, and CD141 (BD Biosciences). The cells were rested at 37°C overnight. Differences in basic phenotype and functional characteristics of mDC1, mDC2, and pDC are listed in Table S1.

In initial studies, moDCs and macrophages were generated by IL-4 and GM-CSF (1 µg/ml) treatment of remaining monocytes following DC isolation. Monocytes were cultured with conditioned medium from KPB-M15 cells, (a gift of Atsunobu Hiraoka, Osaka Dental University, Osaka, Japan) to generate macrophages [56].

### Infection and maturation of DCs

Aliquots of isolated DCs and in vitro-derived moDCs and macrophages (after 7–10 days culture) were exposed to rgRSV. Mock-infected cells were exposed to the same dilution of mock HEp-2 supernatant as used for rgRSV-infected samples. Twenty-four hours post-infection (pi), cells were pelleted, and the supernatants removed and frozen. The cells were stained, fixed in 2% paraformaldehyde and analyzed by flow cytometry. Requirements for divalent cations in RSV infection were examined after EDTA treatment of rgRSV on ice for 30 minutes. DCs were similarly treated with EDTA (30 min room temperature). rgRSV and DCs were then mixed, incubated, and analyzed 24 hr pi. To examine DC maturation, mDCs and pDCs exposed to rgRSV (live, sucrose purified, UV-inactivated, or EDTA-treated) for 24 hrs were stained with the premixed “DC activation reagent” (BD Biosciences), fixed, and analyzed on the FACSCalibur flow cytometer. To evaluate DC maturation in greater detail, a multiparameter flow cytometry panel was developed. RSV-exposed DCs were stained with ViViD viability dye (Molecular Probes) while incubating with Fc block (BD Biosciences), followed by staining with a mixture of phenotypic and maturation cell surface markers. After staining, cells were fixed and analyzed on the LSRII flow cytometer (BD Biosciences). The antibody panels are detailed in Table S2. Flow cytometry data were analyzed using FlowJo software (Tree Star, Inc). Due to donor-to-donor variation in receptor expression levels, mean fluorescence intensities (MFI) for RSV-exposed samples were normalized to mock-infected samples for the same donor to determine the fold-increase in receptor expression. The normalized data from pooled donors were then compared.

### Cytokine and chemokine production

Cytokine and chemokine concentrations in the reserved DC supernatants were measured by ELISA according to kit protocols (R&D Systems). Multiplex cytokine and chemokine analyses were performed on reserved DC supernatants by Aushon Biosystems, Inc. (Billerica, MA). Due to donor-to-donor variation, cytokine and chemokine concentrations (in pg/ml) for RSV-exposed samples were normalized to mock-infected samples for the same donor to determine the fold-increase in protein production. When mock-treated samples were below the level of detection, the protein concentration designated as the limit of detection (as established by the manufacturer's kit protocols) was used for normalization. The normalized data from pooled donors were then compared.

### Statistical Analyses

Statistical comparisons were performed using SigmaStat software. Wilcoxon rank sum analysis of variance was used to determine statistical differences, with significant differences defined where p<0.05. For correlation analyses, Spearman's rank order test was performed. Strong correlations are defined as p<0.01, and weak correlations are defined as 0.01<p<0.05 while positive correlations are demonstrated with r>0 and negative (inverse) correlations with r<0.

## Results

### RSV infects a subset of primary mDC1 and pDCs

**Figure 1:**
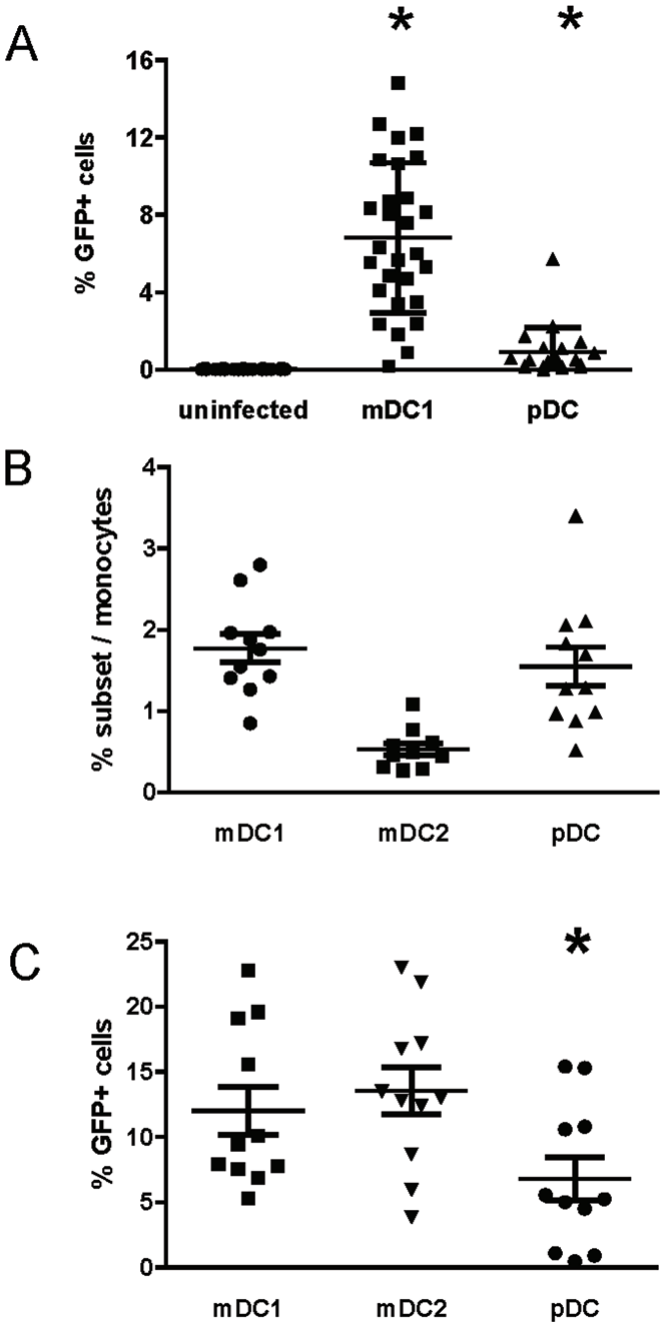
Frequency and RSV infection rates of primary mDC1, mDC2 and pDC. ***Panel A*** – Primary pDC and mDC1 were sequentially isolated from elutriated human monocytes in the first cohort of donors by magnetic selection and infected with rgRSV (moi = 5) as described in the Materials and Methods. The percentage of infected (GFP+) cells was determined 18–24 hrs post-infection for mock-infected cells (•, n = 27), mDC1 (▪, n = 27), and pDCs (▴, n = 20). Relative to mock-infected cells, significantly more mDC1 and pDCs are GFP+ (p<0.0001 and p = 0.007, respectively). ***Panel B*** – In a second cohort of donors, pDC, mDC1, and mDC2 were sequentially isolated from elutriated monocytes. The cell subsets were counted and frequency within the elutriated monocytes calculated. ***Panel C*** – Following magnetic selection and overnight resting, mDC1, mDC2, and pDC were exposed to live rgRSV (moi = 5). Twenty-four hours after infection, uninfected and infected cells were fixed and analyzed by flow cytometry. Data are represented as the percentage of GFP+ cells within live DCs. For panels B and C, N = 11 donor samples with 1 donor present for 2 separate visits. *  =  significantly different from mDC1 and mDC2, p<0.05.

We first determined optimal conditions for RSV infection of primary DCs. Isolated mDC1 and pDCs were infected with varying doses of rgRSV (moi = 1, 3, 5, 10) for 24 hrs. Maximal infection occurred at moi = 5 and did not increase at moi>5 (data not shown). To optimize the kinetics of infection, mDC1 and pDCs were infected with rgRSV (moi = 5), and aliquots of infected cells were analyzed at 0.5, 1, 2, 4, 6, 8, 18–24 (overnight), 48, and 72 hrs post-infection (pi). No infected cells were observed prior to 18–24 hr pi, and the percentage of infected cells did not appreciably increase at 48 or 72 hrs pi (data not shown). Therefore, DC infection and maturation were examined after overnight incubation with RSV (moi = 5). Examination of multiple donors demonstrated that in all donors only a subset of mDC1 and pDCs could be infected by rgRSV (Figure 1A). On average 6.8±3.9% of mDC1 (n = 28 donors, p<0.0001) and 0.9±1.3% of pDCs (n = 20 donors, p = 0.007) were infected with rgRSV at 24 hr pi.

Identification of new phenotypic markers allows the continued division of DCs into functionally distinct subsets, as evidenced by the recent separation of CD11c^+^ mDCs into mDC1 and mDC2 subsets [17]–[20]. Phenotypic and functional markers of mDC1, mDC2, and pDCs are summarized in Table S1. mDC1 and pDCs represent ∼1–3% of cells in the elutriated monocyte fraction while mDC2 are much less frequent with the median mDC2 frequency ∼0.3% (Figure 1B). When infection rates were evaluated in a second cohort of donors, mDC1 and mDC2 infection rates were similar and significantly higher than infection rates in pDCs (Figure 1C).

### RSV exposure increases expression of maturation markers on mDCs and pDCs

**Figure 2:**
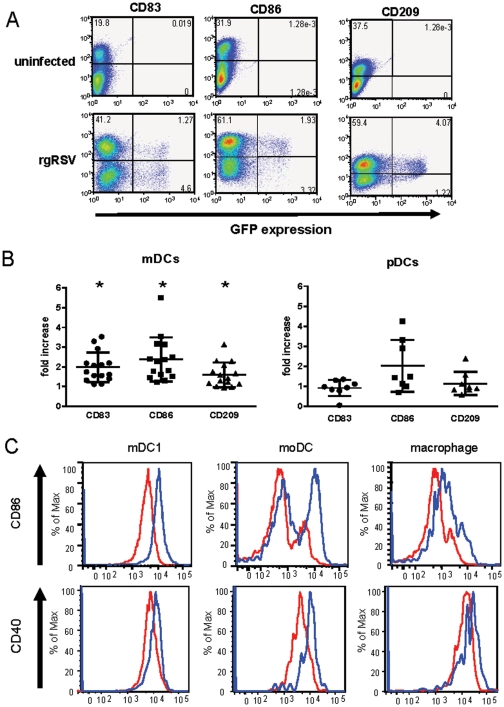
RSV infection and maturation of primary mDC1 and pDCs and of moDCs and macrophages. From elutriated monocytes of a single donor, primary mDC1 and pDC were isolated and moDC and macrophages were generated as detailed in the Materials and Methods. ***Panel A*** – Isolated mDC1 (shown above) and pDC (not shown here) were infected with rgRSV (moi = 5). RSV infection (GFP expression) and DC maturation (CD83, CD86, and CD209 expression) were evaluated after overnight incubation with rgRSV. Dot plot results for mDC1 from a single representative donor (of 15) are shown above. ***Panel B*** – mDC1 and pDCs were exposed to live RSV, and DC maturation was examined 18–24 hrs later. Expression of each maturation marker in mDC1 and pDCs is shown, normalizing the mean fluorescence intensity (MFI) in RSV-exposed DC cultures to mock-infected DCs for the same donor. N = 16 for mDC1 and 8 for pDCs. ***Panel C*** – After overnight exposure of primary mDC1, moDC, and macrophages from a single donor to live rgRSV, DC maturation was examined by flow cytometric analyses of CD86 and CD40 expression. A representative maturation profile is shown with similar maturation patterns observed in 5 independent donors.

Maturation of DCs following exposure to antigenic stimuli results in the upregulation of a number of cell surface proteins. Following RSV infection, expression of CD83, CD86, and, to a lesser extent, CD209 was observed in RSV-infected mDC1 (Figure 2A). The pooled data from 16 mDC1 samples and 8 pDC samples are shown in Figures 2B with each sample obtained from a different donor. While maturation of pDCs was also induced, the magnitude of increased receptor expression was markedly lower than that seen in mDC1 from the same donor. Notably, maturation was induced in both rgRSV-infected and rgRSV–uninfected DCs. This pattern was observed in all donors to varying degrees. DC maturation was due directly to RSV and not other soluble factors (e.g. cytokines) in the viral stock as sucrose-purified virus induced similar levels of DC maturation (data not shown). In pilot studies, we also evaluated mDC1 maturation in comparison to moDC and macrophages differentiated in vitro using monocytes from the same donor (Figure 2C). CD86 and CD40 expression were upregulated to greater extents in moDCs than observed in macrophages or mDC1 (Figure 2C).

### RSV infection induces different patterns of maturation in mDC1, mDC2, and pDCs

**Figure 3:**
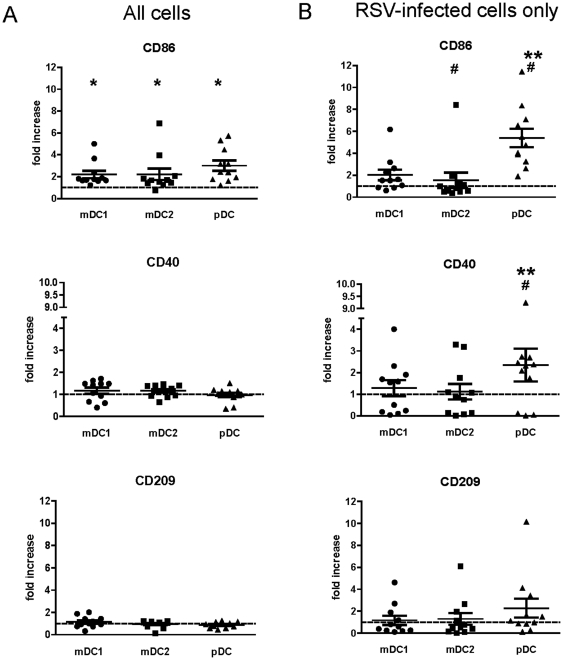
Maturation of RSV-exposed mDC1, mDC2, and pDCs. DC subsets were isolated and infected as detailed in Figure 1. Twenty-four hours after RSV exposure, cells were stained for expression of surface maturation markers and analyzed by flow cytometry. Data are represented as the mean channel fluorescence of RSV-exposed DCs normalized to the mean channel fluorescence of mock-infected cell controls, gating on all live DCs (***panel A***) or on live GFP+ cells only (***panel B***). Actual (non-normalized) MFI values for each donor are detailed in Table S3. N = 11 with 1 donor present for 2 separate visits. *  =  significantly different from mock-infected cell of the same type; **  =  significantly different from mDC1 and mDC2; #  =  significantly different comparing total cells and GFP+ cells only. p<0.05.

In the second donor cohort, maturation of each DC subset was assessed by expression of CD86, CD40, and CD209 following RSV infection. When all cells in the culture were examined, expression of CD86 in RSV-exposed mDC1, mDC2, and pDC was significantly greater than in mock-infected cells (Figure 3A, p = 0.005, p = 0.04, and p = 0.001 for mDC1, mDC2, and pDC, respectively). In contrast, RSV exposure induced little to no increase in CD40 or CD209 expression in the total mDC1, mDC2, or pDC samples (Figure 3A). When expression of maturation markers on RSV-infected (i.e. GFP+) cells was examined (Figure 3B), CD86, CD40, and CD209 upregulation on mDC1 was similar to levels seen in the total RSV-exposed mDC1 pool while CD86 levels on GFP+ mDC2 were significantly lower than levels in the RSV-exposed total culture (p = 0.026). In contrast, CD86, CD40, and CD209 expression on RSV-infected pDCs was greater than on the total pDC pool with the differences in CD86 and CD40 levels attaining statistical significance (p = 0.01 and p = 0.049, respectively). pDCs express lower cell-surface levels of C-type lectins and, therefore, may not be as susceptible to the effects of RSV binding alone in the absence of productive infection. Actual maturation levels (i.e. non-normalized MFIs) on uninfected and infected cells for each donor are detailed in Table S3.

### RSV maturation requires cell surface interaction but not infection

**Figure 4:**
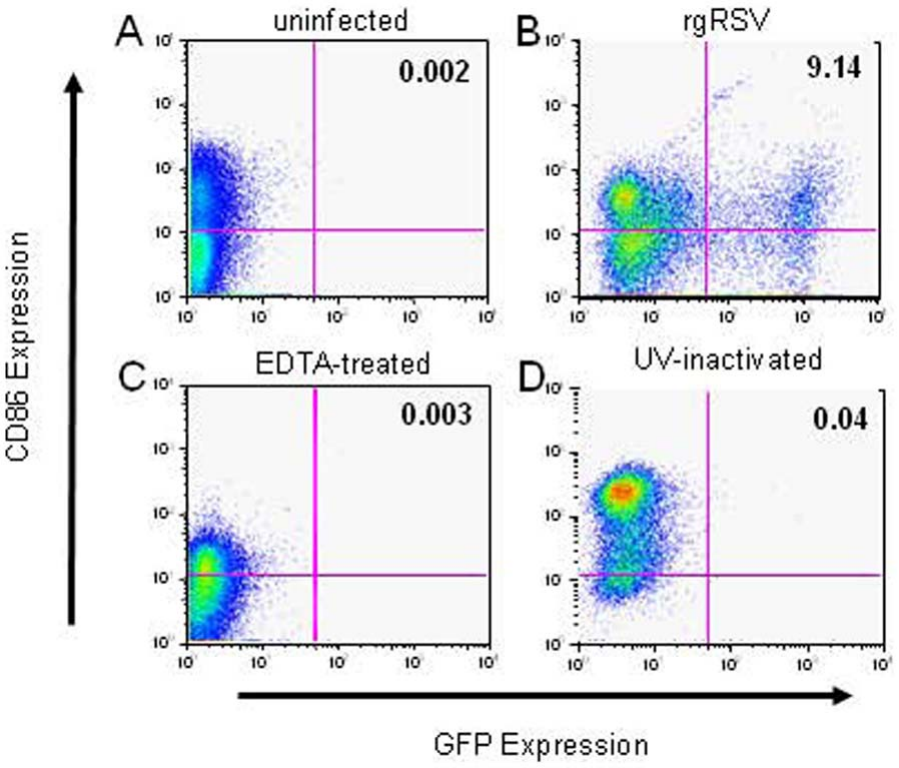
RSV infection of mDC1 requires divalent cations and viral replication, but maturation does not require replication. mDC1 were mock infected (panel A), or exposed to live RSV (panel B), RSV treated with 0.05 M EDTA (panel C), or UV-inactivated RSV (panel D). Equivalent viral particles (moi = 5 of live virus) were used for each RSV exposure. Twenty-four hours after virus exposure the cells were stained for maturation markers (CD83, CD86, CD209) and analyzed by flow cytometry. GFP and CD86 expression is shown in a representative donor (of 8) in which both EDTA-treated and UV-inactivated RSV were used. Data for all donors are summarized in Table 1.

The ability of rgRSV to infect mDC1 (Figure 4) and pDCs (data not shown) was abrogated by pretreatment of the cells or virus with EDTA (Figure 4C) or by UV irradiation of the virus (Figure 4D). Dose response studies demonstrated partial inhibition of infection in the presence of 0.01 M EDTA and full inhibition at 0.05 M EDTA (data not shown). Thus, regardless of the amount of virus used, only a subset of DCs can be infected with RSV, and infection requires the presence of divalent cations. The ability of EDTA treatment to block RSV-induced DC maturation suggests that binding of viral particles to C-type lectins on the cell surface is required for DC maturation. When exposed to UV-inactivated virus, expression of maturation markers was upregulated in mDC1 (Figure 4D) and pDCs (data not shown). Increased expression occurred in the complete absence of GFP expression, demonstrating RSV replication is not required for DC maturation. Representative data in Figure 4 shows changes in GFP (infection) and CD86 expression (maturation) for a single donor. Similar patterns of maturation and inhibition were seen for all phenotypic markers and in all donors. The pooled data for these treatments of RSV-infected mDC1 are summarized in Table 1. Although upregulation of maturation markers was triggered by exposure to UV-inactivated rgRSV, the level of maturation marker expression was generally less than that seen with exposure to live rgRSV (Table 1). These data suggest that RSV induces DC maturation both by interaction of virus particles with the cell surface and by processes associated with viral entry and/or replication.

**Table 1:** Maturation of mDCs After RSV Infection.

	# of Donors	CD83	CD86	CD209
Uninfected	27	1±0	1±0	1±0
rgRSV	28	1.98±0.76^a^	2.38±1.12^a^	1.60±0.62^a^
EDTA-treated rgRSV	8	1.34±0.47^b^	1.33±0.73^b^	0.99±0.24^b^
UV-inactivated rgRSV	6	1.21±0.32^b^	1.79±0.46^a^	1.27±0.30

Data are represented as the fold increase in mean channel fluorescence (MFI) relative to the uninfected cells for each individual donor.

a significantly different than uninfected cells, p<0.05.

b significantly different than rgRSV cells, p<0.05.

### RSV infection induces different patterns of cytokine and chemokine production in mDC1, mDC2, and pDCs

**Figure 5:**
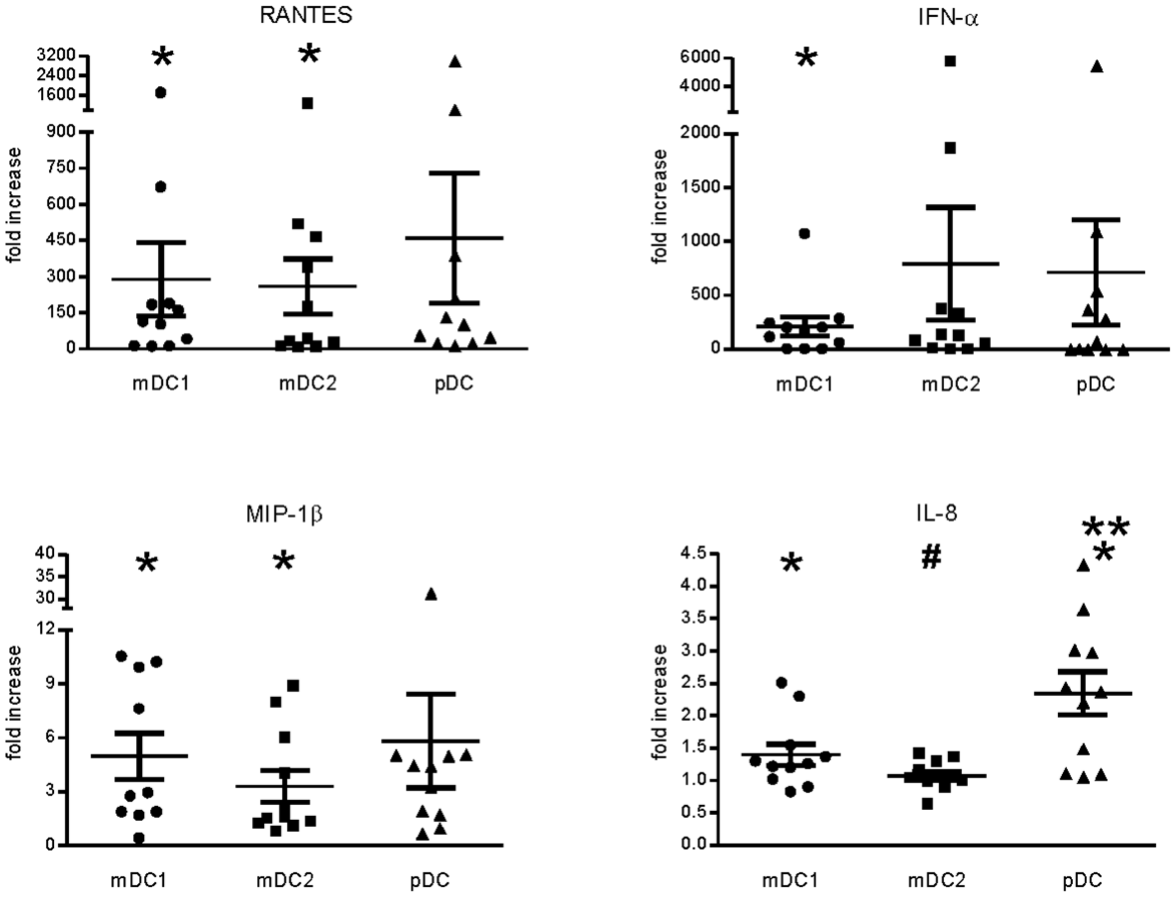
Cytokine and chemokine production as measured by standard sandwich ELISA. DC subsets were isolated and exposed to RSV as described in Figure 1. Twenty-four hours after RSV infection, the culture supernatants were removed and frozen. Subsequently, cytokine and chemokine levels were measured by sandwich ELISA according to kit protocols. Data represent production levels of RSV-exposed DCs normalized to uninfected DCs of the same cell type. Actual (non-normalized) cytokine concentrations for each donor are detailed in Table S3. N = 11 with 1 donor present for 2 separate visits. *  =  significantly different from mock-infected cells of the same type; **  =  significantly different from mDC1 and mDC2; #  =  significantly different from mDC1 only. p<0.05.

Secretion of cytokines and chemokines are also triggered as a result of DC activation. Production of RANTES, IL-6, IL-8, MIP-1α, MIP-1β, and IFN-α by RSV-exposed DCs were examined by traditional sandwich ELISA (Figure 5 and Table S3). Our preliminary studies of RSV infection of primary mDC1 and pDC demonstrated no detectable production of IFN-β, IFN-γ, TNF-α, or IL-12. Therefore, these cytokines were not examined in this set of donors. RSV exposure induced production of each cytokine in most donors, although increases in IL-6 levels of RSV-exposed cells were not significantly different than levels in mock-treated cells for any DC subset (Table S3). In contrast, all other cytokines were significantly increased in RSV-exposed mDC1 (Figure 5). RANTES and MIP-1β levels in RSV-exposed mDC2 were significantly greater than in mock-treated cells, while increases in MIP-α and IFN-α were not (Figure 5 and Table S3). RSV infection of mDC2 produced little increase above basal production of IL-8 in most donors (Figure 5 and Table S3). While production of all cytokines were increased in pDCs following RSV exposure, only IL-8 levels in RSV-exposed pDC were significantly greater than in mock-treated cells (Figure 5, p = 0.002).

When comparing the induction of cytokine and chemokine responses across DC subsets, only IL-8 production showed significant differences. IL-8 production by pDCs was significantly greater than in RSV-exposed mDC1 or mDC2 (Figure 5, p = 0.009 and 0.0006, respectively). Additionally, increases in mDC1 IL-8 levels were greater than in mDC2 (p = 0.039). Differences in the induction of RANTES, MIP-1α, MIP-1β, and IFN-αbetween DC subsets were not statistically significant. While IFN-α was induced in all DC subsets, the absolute concentrations of IFN-α secreted by mDC1 and mDC2 were markedly lower than levels in pDC from the same donor (Table S3). Cytokine concentrations from individual donors for each uninfected and infected DC subset are listed in detail in Table S3.

**Figure 6:**
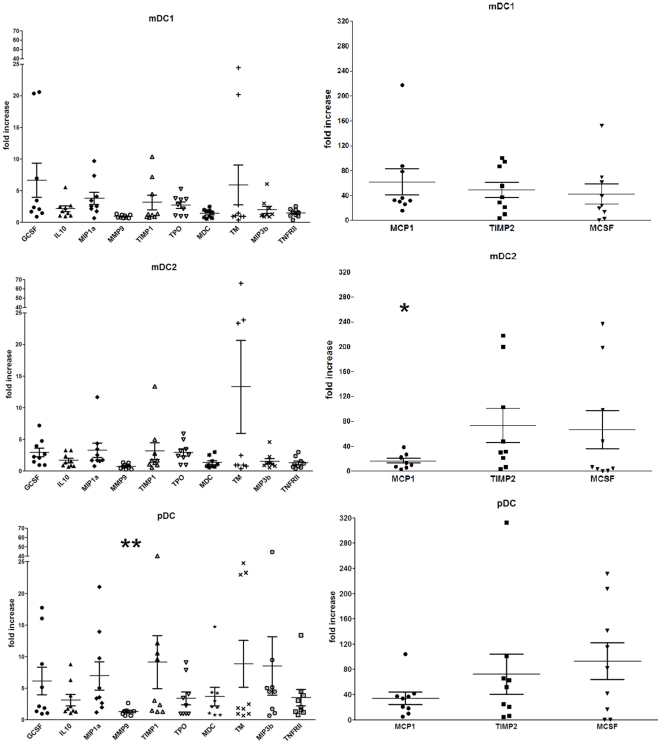
RSV-induced of cytokine, chemokine, and growth factor production measured by multiplex analyses. Cytokine and chemokine levels were measured by multiplex assay. Data represent production levels of RSV-exposed DCs normalized to uninfected DCs of the same cell type. Actual (non-normalized) cytokine concentrations for each donor are detailed in Table S3. N = 9 unique donors. *  =  significantly different from mDC1 only; **  =  significantly different from mDC1 and mDC2, p<0.05.

Analyses of cytokine, chemokine, and growth factor production were extended by multiplex analysis (Figure 6). The panel of analytes included IL-8, IL-10, IL-12, G-CSF, M-CSF, MIP-1α, MIP-3β, monocyte chemoattractant protein-1 (MCP-1), macrophage-derived chemokine (MDC), matrix metalloproteinase-9 (MMP-9), tissue inhibitor of MMP 1 (TIMP-1), TIMP-2, thrombopoietin (TPO), thrombomodulin (TM), and tumor necrosis factor receptor type 2 (TNF-RII). IL-12 production was not detected in any uninfected or RSV-infected sample from any donor (Table S3). When production was normalized to uninfected cells of the same subset, levels of most other analytes were significantly increased (Figure 6 and Table S3). TPO, M-CSF, and TM were generally not detectable in uninfected mDC1, mDC2, or pDC, but were induced following RSV exposure, so the protein concentration defined as the limit of detection was used for the mock-infected value in normalization of these chemokines. In RSV-exposed mDC1, IL-8, IL-10, G-CSF, M-CSF, MIP-1α, MIP-3β, MCP-1, MDC, TIMP-1, TIMP-2, TPO, and TNF-RII were significantly increased relative to mock-treated cells while changes in MMP-9 and TM production were not significantly different (Figure 6 and Table S3). In contrast, RSV-exposed mDC2 exhibited lower fold increases in production with significant differences occurring only in G-CSF, IL-10, MCP-1, MIP-1α, TIMP-2, TPO, and M-CSF (Figure 6). While the fold-change in cytokine and chemokine levels in mDC2 were often lower than the normalized values in mDC1, mDC2 have inherently higher levels of baseline production (Table S3 and Figure S1). Therefore, on the basis of absolute values, RSV-exposed mDC2 had comparable post-infection cytokine and chemokine levels as mDC1 (Table S3). As with mDC1, RSV induced marked increases in most of the measured cytokines and chemokines in pDCs with statistically significant differences observed in IL-8, IL-10, G-CSF, M-CSF, MIP-1α, MCP-1, MDC, TIMP-1, TIMP-2, TPO, TM, and TNF-RII (Figure 6 and Table S3). MMP-9 and MIP-3β were the only analytes not significantly increased by RSV exposure of pDCs.

In all three DC subsets, two distinct patterns of induction were apparent. Most analytes had a relatively low level of induction (≤10-fold) while a small group of analytes (including MCP-1, TIMP-2, and M-CSF) had high levels (>10-fold) of induction (Figure 6). While the general pattern of “low” and “high” responses occurred for the same analytes across the DC subsets, cell-specific patterns of cytokine and chemokine production were observed within these groups. TIMP-1 production was generally greater in pDC than in mDC1 or mDC2. In contrast, increases in TIMP-2 and M-CSF were similar in pDC and mDC2 and were greater than those seen in mDC1. MCP-1 levels were greater in mDC1 than in mDC2 or pDC. Additionally, while changes in MMP-9 levels were not statistically significant comparing mock-treated and RSV-exposed DCs for each subset, significant differences were observed between the primary DC subsets with pDCs having greater increases in expression (normalized level  = 1.34) relative to mDC1 (0.97) and mDC2 (0.78). Absolute cytokine and chemokine levels from uninfected and infected cultures for each donor are listed in Table S3.

### Identification of potential cell-specific response patterns in RSV-exposed DCs

Variations were observed between donors in the rates of RSV infection (i.e. GFP expression), in the upregulation of maturation markers, and in cytokine and chemokine production. Therefore, the data were analyzed by Spearman's rank order correlation to determine if associations exist between the susceptibility to RSV infection, the magnitude of DC maturation, or the production of cytokines after RSV exposure. These analyses are detailed in Table S4. Correlation analyses demonstrated no correlation between infection rates and expression of maturation markers or cytokine production in mDC1 and pDC with only a weak correlation between pDC infection rates and TIMP-1 production observed. When upregulation of maturation markers and cytokine/chemokine production were compared, positive correlations were found between CD86 and CD40 expression on mDC2 and between CD86 expression and MMP9 production in pDCs. In contrast, CD86 expression on mDC1 was negatively correlated with production of MIP-1β while CD209 expression on mDC2 was inversely associated with M-CSF production.

Correlation analyses of cytokine and chemokine production within each DC subset were also performed and are detailed in Tables S5, S6, and S7 for mDC1, mDC2, and pDC, respectively. Comparison of the correlations between each DC subset suggests a potential “myeloid signature” of cytokine and chemokine production with the strong positive correlations in mDC1 and mDC2 between IL-6, IL-8, and G-CSF and TIMP-1 and TPO (denoted by black framed values) while no correlation is found between these cytokines in RSV-exposed pDCs. Unique to mDC1, inverse correlations were found between TM and MIP-1α and MIP-1β. MMP9 production was found in strong positive correlation to production of IL-6, IL-8, G-CSF, TIMP-1, TPO, and MDC in mDC2 (red framed values). In pDCs only were MMP9, TIMP-1, and TPO associated with MIP-1α production and M-CSF with IL-6, G-CSF, and MMP9, although the negative correlations were weak (0.01<p<0.05). This report is the first examination of MMP9, TIMP-1, and TIMP-2 production in human DCs during RSV infection. The correlation of MMP9 and TIMP-1 with the production of a number of cytokines potentially suggests functional roles for these mediators in RSV-induced immunity. Furthermore, identification of distinct associations of these cytokines among the DC subsets and the lack of correlation between TIMP-2 with any other cytokine (despite high levels of TIMP-2 production) may indicate RSV- and cell-specific effects for the chemokines within mDC1, mDC2, and pDC subsets. Further examination of such cytokine and chemokine production profiles may definitively identify subset-specific patterns which can subsequently reveal distinctive functional roles for individual DC subsets during RSV infection.

## Discussion

Dendritic cells are critical for initiation and imprinting of immune responses. Both mDCs and pDCs are recruited to the respiratory tract in virally-infected infants, and differences in DC recruitment and function have been observed in children who experienced bronchiolitis compared to mild upper respiratory infection or in infants infected with RSV compared to those infected with influenza [41]–[43]. RSV is often the first viral infection in life. Because the initial exposure to RSV antigens during primary infection of infants may play a role in establishing susceptibility to subsequent reinfection, it is important to understand the effects of RSV exposure on the maturation and function of primary human mDCs and pDCs. In this report we have used GFP-expressing RSV A2 and multi-parameter flow cytometric analyses to examine the impact of RSV infection on primary mDCs and pDCs. We demonstrate that both mDCs and pDCs are infected with RSV in a divalent cation-dependent manner. While variation in infection rates among donors was observed, mDC1 and mDC2 infection rates were greater than that of pDCs. Additionally, exposure to RSV induced maturation of both infected and uninfected mDCs and pDCs as previously observed in moDCs [33]. Similarly, RSV stimulated the production of cytokines from mDCs and pDCs. This was particularly true for RANTES secretion (Figure 5) which has not been previously reported from RSV-infected primary DCs. In contrast, IL-10, IL-12, IFN-γ, and TNF-α have been reported in RSV-infected cord-blood-derived DCs and moDCs [29], [34], [37]–[39], but no IL-12 and only very low levels of IL-10 were detected in RSV-exposed primary mDCs or pDCs in this study (Figure 6 and Table S3). A caveat is that we used a recombinant virus based on the A2 strain of RSV. A prior report using RSV Long indicated that mDCs produced IL-12, although the data were not shown [44]. Additionally, basal levels of IL-6 and IL-8 (i.e. in mock-infected cells) were higher than expected. This may reflect some level of cellular activation during monocyte elutriation. Alternatively, this may also represent low level acute activation resulting from the very short (overnight) incubation of the isolated DC subsets on plastic in the presence of GM-CSF or IL-3 – which has been shown to be sufficient to initiate DC activation and induces formation of dendritic processes visible by light microscopy (data not shown). While these cytokines are present, there is not significant surface expression of traditional activation markers such as CD80, CD86, and CD40. Therefore, if activation during the processing phase has occurred, it is minimal.

UV-inactivated virus was able to induce maturation without viral replication, although increases in maturation markers and cytokine production did not attain the maximal levels induced by live RSV infection of the same donor (Table 1). These data indicate RSV binding may be sufficient to mediate at least partial DC activation. However, divalent cations were required for both infection and maturation, suggesting the interaction of RSV surface proteins with C-type lectins on DCs transmits signals that affect maturation independent of infection. While not identified by reconstitution experiments in our study, the divalent cation critically required for RSV infection of DCs presumably is Ca^2+^ which has been demonstrated to be necessary for RSV infection of [57] and syncytium formation in HEp-2 cells [58] and for RSV F and G binding to surfactant proteins [59], [60]. It is clear from the pDC data that infection causes a different level of activation that is distinct from just binding of surface receptors. In pDCs, CD40 expression in 5 donors (of 11) and CD209 expression in 7 donors (of 11) is decreased in RSV-exposed cells (normalized value <1). This is in striking contrast to the expression of maturation markers on productively infected, GFP+ pDCs where marked increases in CD40 and CD209 expression and significantly higher expression of CD86 relative to total RSV-exposed pDCs are observed (Figure 6). This is similar to findings of Shingai et. al. in which RSV inhibition of IFN-β production in transfected HEK293 and moDCs was shown to occur in two phases, one dependent upon virus-cell contact with infection and another interaction mediated by soluble RSV G [61]. These findings may explain why pDCs are less likely to be infected, but more dependent on infection to trigger the maturation process. The decrease in pDC infectivity may also reflect an inhibition of replication due to the increased production of IFN-α observed in these cells. Therefore, we suggest a two-step process is involved in maximal activation of primary human DCs by RSV – one which is triggered by the binding of viral particles and a second initiated by viral entry and/or replication, and future studies of RSV and DC interactions should attempt to discriminate the signaling events induced by attachment and binding from the events associated with entry and viral replication.

Studies have shown a lack of DC activation following exposure to UV-inactivated RSV [31], [33], [34], [37], [39]. However, our data (Figure 4) and several previous reports [28], [29], [40], [44], [62] demonstrate UV-inactivated RSV can induce DC activation or impact DC function in the absence of viral replication. UV irradiation can induce structural changes resulting in loss of protein function [63], [64] with sensitivity to UV inactivation varying among different proteins [65]. As UV inactivation may disrupt disulfide cysteine bonds [66], the conserved cysteine noose of RSV G and the cysteine-rich domain between the heptad repeats of RSV F potentially make the two major surface viral glycoproteins particularly sensitive to UV treatment. Thus, the disparate reports on DC activation by UV-exposed RSV may be explained by the multiple methods used to inactivate the virus.

While levels of maturation markers such as CD83 and CD86 increased on both infected and uninfected mDCs and pDCs (Figures 2 and 3), CD40 expression was upregulated only on RSV-infected pDCs (Figure 3). The failure to upregulate CD40 on mDCs and uninfected pDCs may be relevant to the subsequent ability of RSV-exposed DCs to initiate or expand virus-specific adaptive immunity. CD40 is critical for co-stimulation and activation of antigen-specific T and B cells [67]. CD40 expression on DCs may also influence activation and function of NK and NKT cells [68], [69] which can impact RSV pathogenesis in the mouse model [70].

Potential roles for MMP-9 and its inhibitor TIMP-1 in RSV pathogenesis have been reported. RSV was shown to induce MMP-9 expression in cultured epithelial cell lines and to be involved in syncytium formation by infected cells [57]. Furthermore, transfection of cells with TIMP-1 prior to RSV infection reduced syncytium size [57]. These studies were extended by Elliott et al. with the demonstration of MMP-9 and TIMP-1 in the nasopharyngeal secretions of infants infected with RSV or parainfluenza virus with levels correlating with disease severity as assessed by hypoxia [71]. However, while MMP-9 and TIMP-1 were produced by RSV-infected transformed cells lines (e.g. A549), they were not detected in RSV-infected primary normal human bronchial epithelia (NHBE). TIMP-2 production was not detected in either paramyxovirus-infected cell lines or NHBE. While RSV infection of DCs did not increase MMP-9 production (Figure 6), we demonstrate for the first time that RSV infection induces marked increases of TIMP-2 and (lower levels of increases in TIMP-1) in all DC subsets. Thus, DCs may be an important source of TIMP-2, and could thereby contribute to the regulation of MMP-9 in the respiratory tract.

Innate immunity at mucosal surfaces such as the lung is highly complex and flexible, maintaining the balance between tolerance and inflammation while under constant insult from environmental factors. This may be reflected in the diversity of DC phenotypes found at the mucosa of specific tissues and within different compartments of a single organ [72], [73]. DC precursors and immature DCs in the lung respond to factors secreted by resident epithelial and innate cells, resulting in differentiation and activation [72]–[76]. Monocytes residing in the respiratory tract have been suggested as a local precursor for pulmonary DCs, particularly maintaining homeostatic balance [72], [73], [75]. Studies in mice have demonstrated that blood monocytes subsets have the potential to differentiate into distinct subsets of respiratory tract DCs [77], [78]. However, these studies do not exclude a role for blood-derived DCs in pulmonary inflammation, and the rapidity of DC infiltration in response to local antigen challenge [79] suggests that recruitment of DCs from blood can be a rich source of antigen presenting cells. Thus, achieving the level of functional and phenotypic DC diversity in distinct compartments of the respiratory tract under normal versus inflammatory conditions may require both differentiation of local monocytes and recruitment from the blood of immature or precursor mDCs and pDCs.

In this detailed analyses of primary mDC1, mDC2, and pDC from a relatively large number of donors we demonstrate that RSV has distinct effects on maturation and cytokine production in each subpopulation of primary human DCs. All RSV-exposed DCs have increased CD86, but only RSV-infected pDCs upregulate CD40 and CD209. mDC1 express lower levels of TIMP-2 and M-CSF, but have greater production of MCP-1 after RSV exposure than mDC2 or pDC. IL-8 production and CD40 expression on RSV-infected mDC2 was minimal, but levels of TIMP-2 and M-CSF were greater than in mDC1 and comparable with pDC production. Furthermore, our data indicate that TIMP-2 is produced by RSV-exposed DCs, suggesting a potential role for DCs in regulating the effects of MMPs induced during RSV infection. Understanding these interactions is important for defining new therapeutic approaches for severe RSV disease. Future studies should take into account potential differences in RSV strain-specific interactions with human DCs, which would require construction of additional molecular clones of RSV or alternative methods for detecting individual RSV-infected cells. Considering that RSV is often the first virus encountered by neonates, defining the role of RSV in DC biology may be important for understanding disease pathogenesis and may have broader implications for lung development and maturation of the human immune system.

## Supporting Information

Figure S1 **Basal levels of cytokine, chemokine, and growth factor production measured by multiplex analyses.** mDC1, mDC2, and pDC were isolated and exposed to RSV as described in Figure 3. Twenty-four hours after RSV infection, the culture supernatants were removed and frozen. Subsequently, cytokine and chemokine levels were measured by multiplex assay. Data represent basal levels of mock-infected DCs as protein concentration in pg/ml standardized to 10^5^ cells. N = 9 unique donors.
(TIFF)

Table S1 **Phenotype and Functional Comparison of Primary Human DC Subsets.** The standard cell surface markers used for differentiation of mDC1, mDC2, and pDC and the main functions of each DC subset are detailed.
(TIFF)

Table S2 **Multiparameter Flow Cytometry Panels.**
(TIFF)

Table S3 **Individual Values for Donor Set #2.** The values for infection rates, mean fluorescence intensity of cell surface markers, and levels of cytokines produced are shown for mock- and RSV-infected mDC1, mDC2, and pDC for each individual donor. Cytokine values represent the concentration of each cytokine in pg/ml at 1×10^5^ cells per tube after overnight infection. NT  =  not tested. ND  =  not detected.
(TIF)

Table S4 **Correlation Analyses (Spearman's Rank Order) of RSV Infection Rates with Maturation and Cytokine/Chemokine Production.** For the RSV-exposed DCs, infection rates were correlated with expression of maturation markers and with cytokine and chemokine production for each donor. Maturation marker expression was also correlated with cytokine and chemokine levels for each donor. These analyses were performed using Spearman's rank order test of correlation. Strong positive correlations are defined as r>0 and p<0.01 and are denoted by bold red values in gray shaded boxes while weak positive correlations are defined as r>0 and 0.01<p<0.05 and are denoted by bold red values. Similarly, strong negative correlations are defined as r<0 and p<0.01 and are denoted by bold blue values in gray shaded boxes while weak negative correlations are defined as r>0 and 0.01<p<0.05 and are denoted by bold blue values.
(TIF)

Table S5 **Correlation Analyses (Spearman's Rank Order) of mDC1 Cytokine/Chemokine Production.** For each individual donor (data detailed in Table S3), cytokine and chemokine production by RSV-exposed mDC1 were correlated to each other. These analyses were performed using Spearman's rank order test of correlation. Strong positive correlations are defined as r>0 and p<0.01 and are denoted by bold red values in gray shaded boxes while weak positive correlations are defined as r>0 and 0.01<p<0.05 and are denoted by bold red values. Similarly, strong negative correlations are defined as r<0 and p<0.01 and are denoted by bold blue values in gray shaded boxes while weak negative correlations are defined as r>0 and 0.01<p<0.05 and are denoted by bold blue values.
(TIF)

Table S6 **Correlation Analyses (Spearman's Rank Order) of mDC2 Cytokine/Chemokine Production.** For each individual donor (data detailed in Table S3), cytokine and chemokine production by RSV-exposed mDC2 were correlated to each other. These analyses were performed using Spearman's rank order test of correlation. Strong positive correlations are defined as r>0 and p<0.01 and are denoted by bold red values in gray shaded boxes while weak positive correlations are defined as r>0 and 0.01<p<0.05 and are denoted by bold red values. Similarly, strong negative correlations are defined as r<0 and p<0.01 and are denoted by bold blue values in gray shaded boxes while weak negative correlations are defined as r>0 and 0.01<p<0.05 and are denoted by bold blue values.
(TIF)

Table S7 **Correlation Analyses (Spearman's Rank Order) of pDC Cytokine/Chemokine Production.** For each individual donor (data detailed in Table S3), cytokine and chemokine production by RSV-exposed pDC were correlated to each other. These analyses were performed using Spearman's rank order test of correlation. Strong positive correlations are defined as r>0 and p<0.01 and are denoted by bold red values in gray shaded boxes while weak positive correlations are defined as r>0 and 0.01<p<0.05 and are denoted by bold red values. Similarly, strong negative correlations are defined as r<0 and p<0.01 and are denoted by bold blue values in gray shaded boxes while weak negative correlations are defined as r>0 and 0.01<p<0.05 and are denoted by bold blue values.
(TIF)
