# Supplementary figures and images for: Primary Human mDC1, mDC2, and pDC Dendritic Cells Are Differentially Infected and Activated by Respiratory Syncytial Virus
Source: PLoS One. 2011 Jan 28;6(1):e16458. doi: 10.1371/journal.pone.0016458 (PMC3030580; doi:10.1371/journal.pone.0016458)

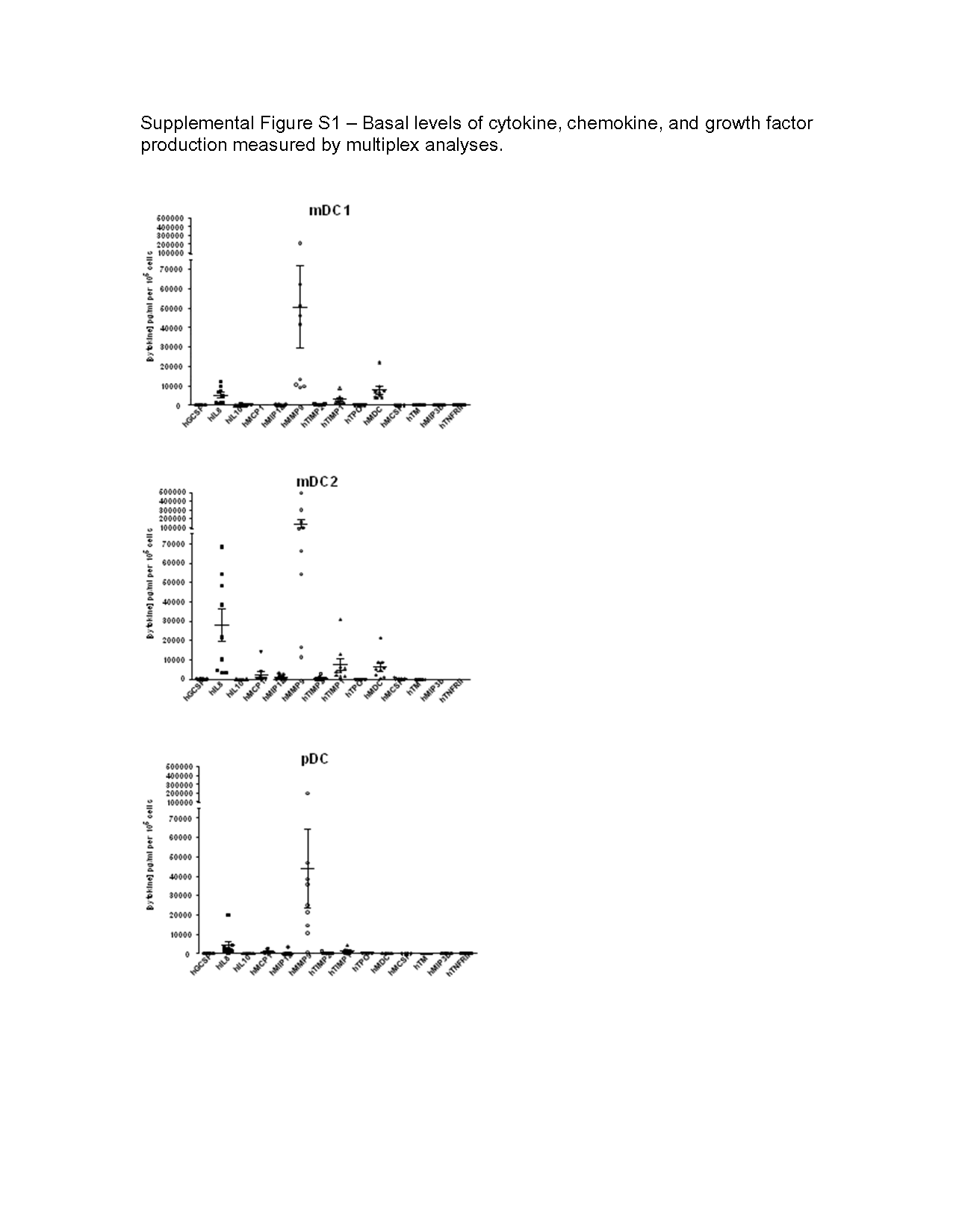

Supplement: Figure S1 — Basal levels of cytokine, chemokine, and growth factor production measured by multiplex analyses. mDC1, mDC2, and pDC were isolated and exposed to RSV as described in Figure 3. Twenty-four hours after RSV infection, the culture supernatants were removed and frozen. Subsequently, cytokine and chemokine levels were measured by multiplex assay. Data represent basal levels of mock-infected DCs as protein concentration in pg/ml standardized to 105 cells. N = 9 unique donors. (TIFF) [file pone.0016458.s001.tiff]

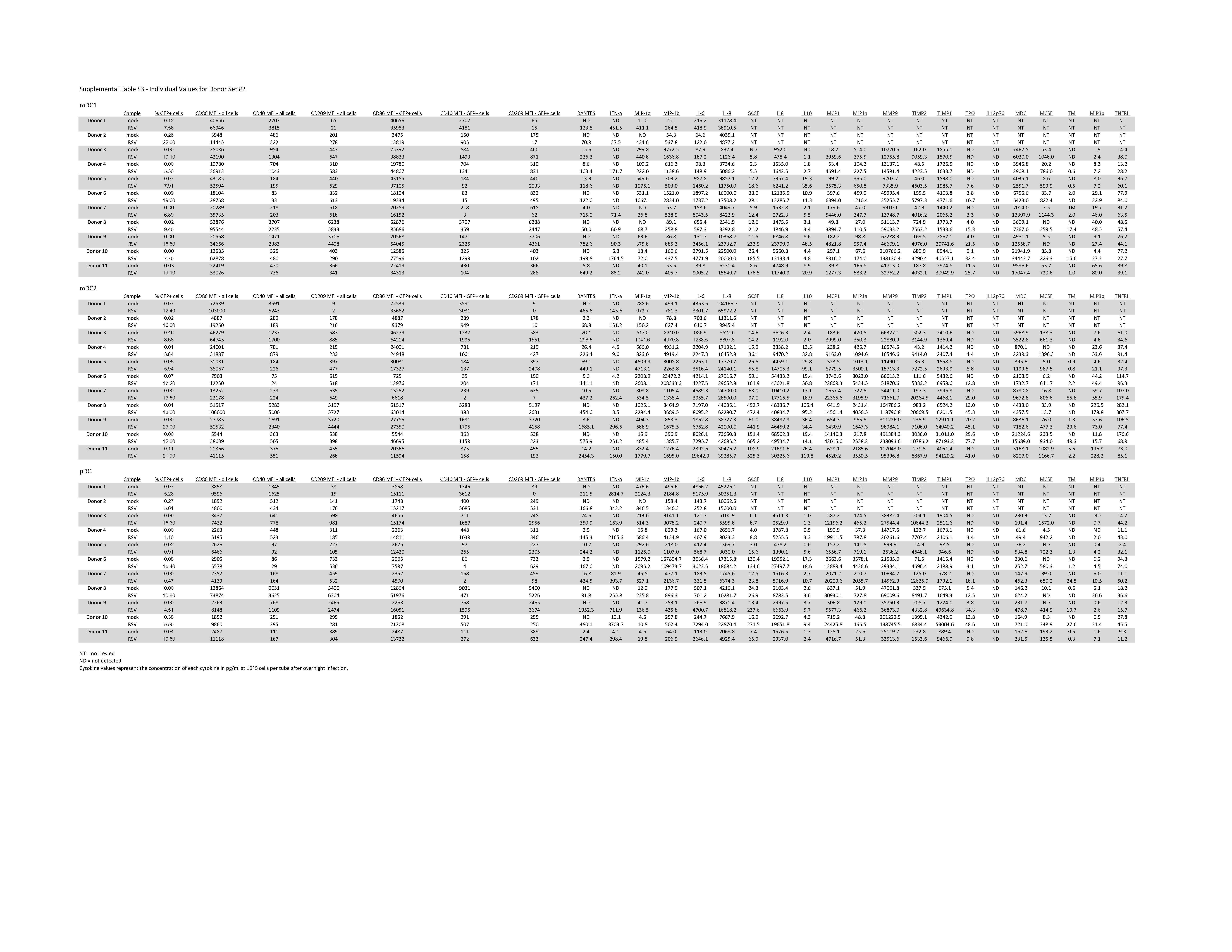

Supplement: Table S3 — Individual Values for Donor Set #2. The values for infection rates, mean fluorescence intensity of cell surface markers, and levels of cytokines produced are shown for mock- and RSV-infected mDC1, mDC2, and pDC for each individual donor. Cytokine values represent the concentration of each cytokine in pg/ml at 1×10^5^ cells per tube after overnight infection. NT = not tested. ND = not detected. (TIF) [file pone.0016458.s004.tif]

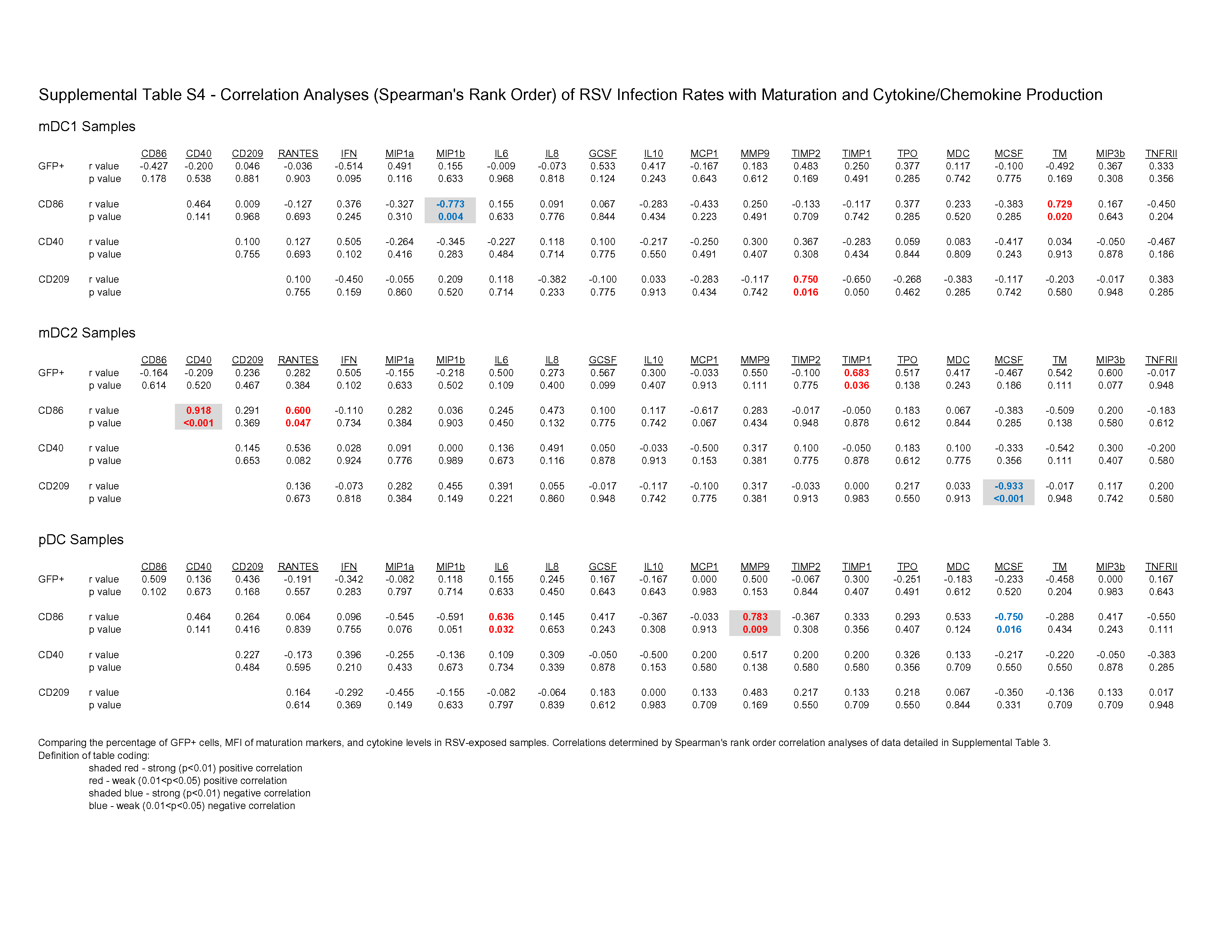

Supplement: Table S4 — Correlation Analyses (Spearman's Rank Order) of RSV Infection Rates with Maturation and Cytokine/Chemokine Production. For the RSV-exposed DCs, infection rates were correlated with expression of maturation markers and with cytokine and chemokine production for each donor. Maturation marker expression was also correlated with cytokine and chemokine levels for each donor. These analyses were performed using Spearman's rank order test of correlation. Strong positive correlations are defined as r>0 and p<0.01 and are denoted by bold red values in gray shaded boxes while weak positive correlations are defined as r>0 and 0.01<p<0.05 and are denoted by bold red values. Similarly, strong negative correlations are defined as r<0 and p<0.01 and are denoted by bold blue values in gray shaded boxes while weak negative correlations are defined as r>0 and 0.01<p<0.05 and are denoted by bold blue values. (TIF) [file pone.0016458.s005.tif]

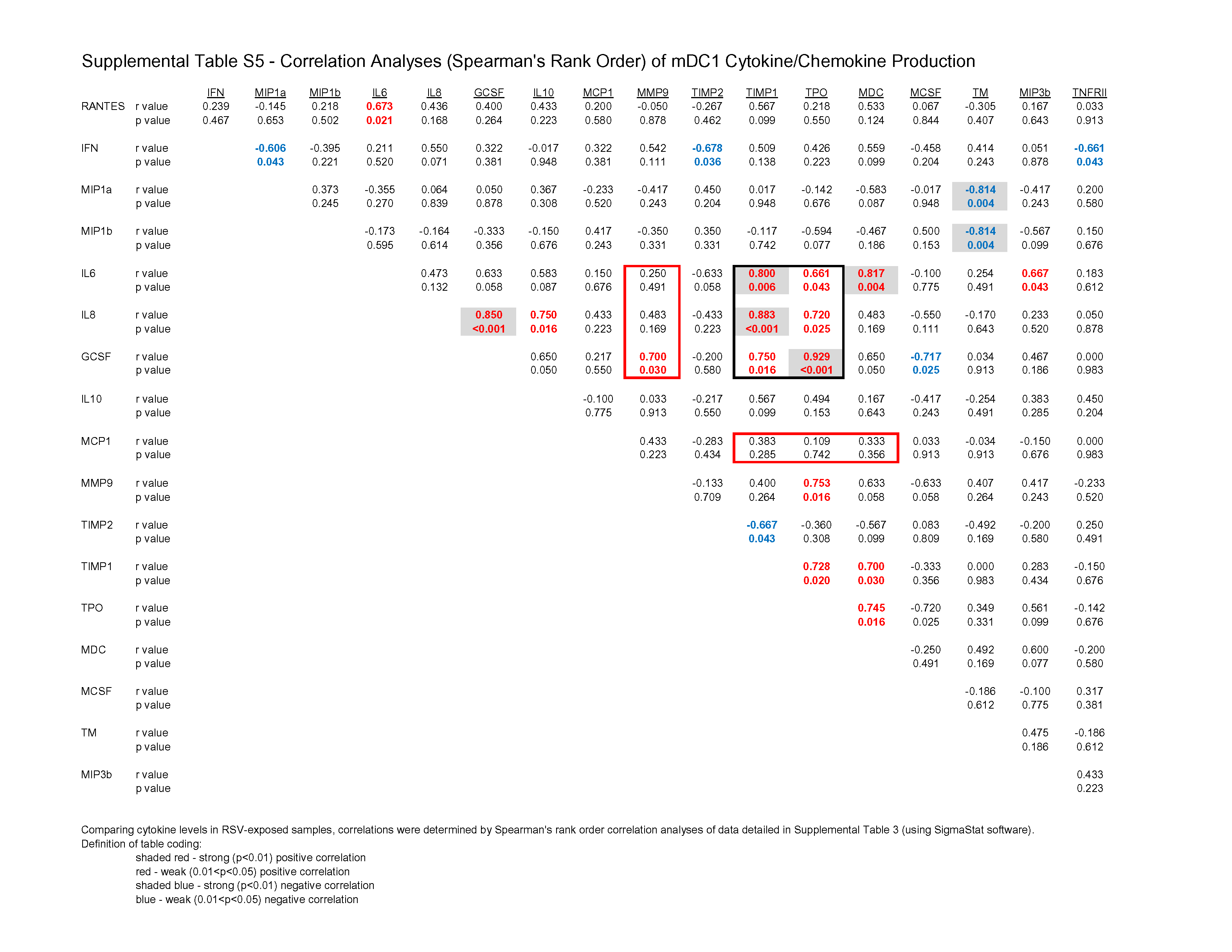

Supplement: Table S5 — Correlation Analyses (Spearman's Rank Order) of mDC1 Cytokine/Chemokine Production. For each individual donor (data detailed in Table S3), cytokine and chemokine production by RSV-exposed mDC1 were correlated to each other. These analyses were performed using Spearman's rank order test of correlation. Strong positive correlations are defined as r>0 and p<0.01 and are denoted by bold red values in gray shaded boxes while weak positive correlations are defined as r>0 and 0.01<p<0.05 and are denoted by bold red values. Similarly, strong negative correlations are defined as r<0 and p<0.01 and are denoted by bold blue values in gray shaded boxes while weak negative correlations are defined as r>0 and 0.01<p<0.05 and are denoted by bold blue values. (TIF) [file pone.0016458.s006.tif]

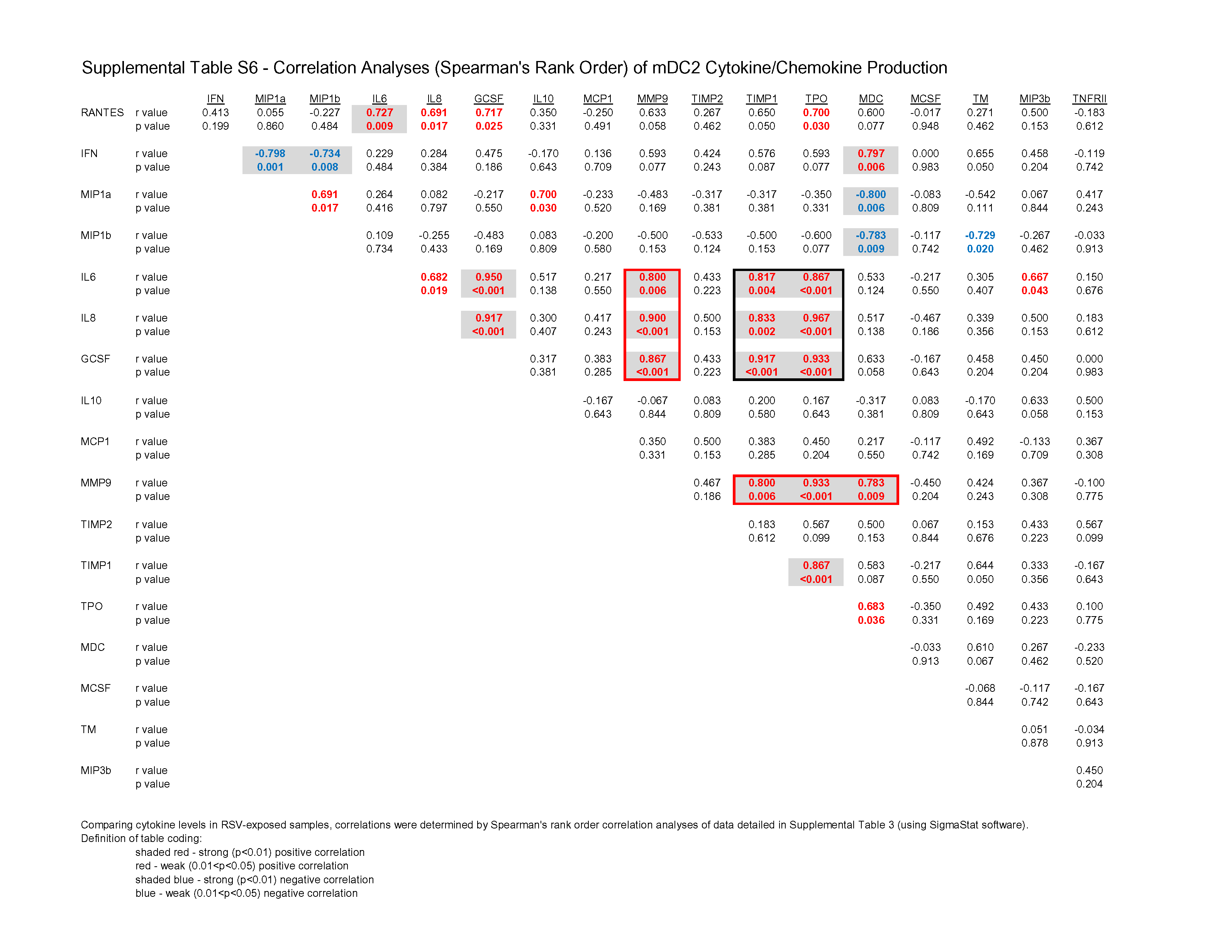

Supplement: Table S6 — Correlation Analyses (Spearman's Rank Order) of mDC2 Cytokine/Chemokine Production. For each individual donor (data detailed in Table S3), cytokine and chemokine production by RSV-exposed mDC2 were correlated to each other. These analyses were performed using Spearman's rank order test of correlation. Strong positive correlations are defined as r>0 and p<0.01 and are denoted by bold red values in gray shaded boxes while weak positive correlations are defined as r>0 and 0.01<p<0.05 and are denoted by bold red values. Similarly, strong negative correlations are defined as r<0 and p<0.01 and are denoted by bold blue values in gray shaded boxes while weak negative correlations are defined as r>0 and 0.01<p<0.05 and are denoted by bold blue values. (TIF) [file pone.0016458.s007.tif]

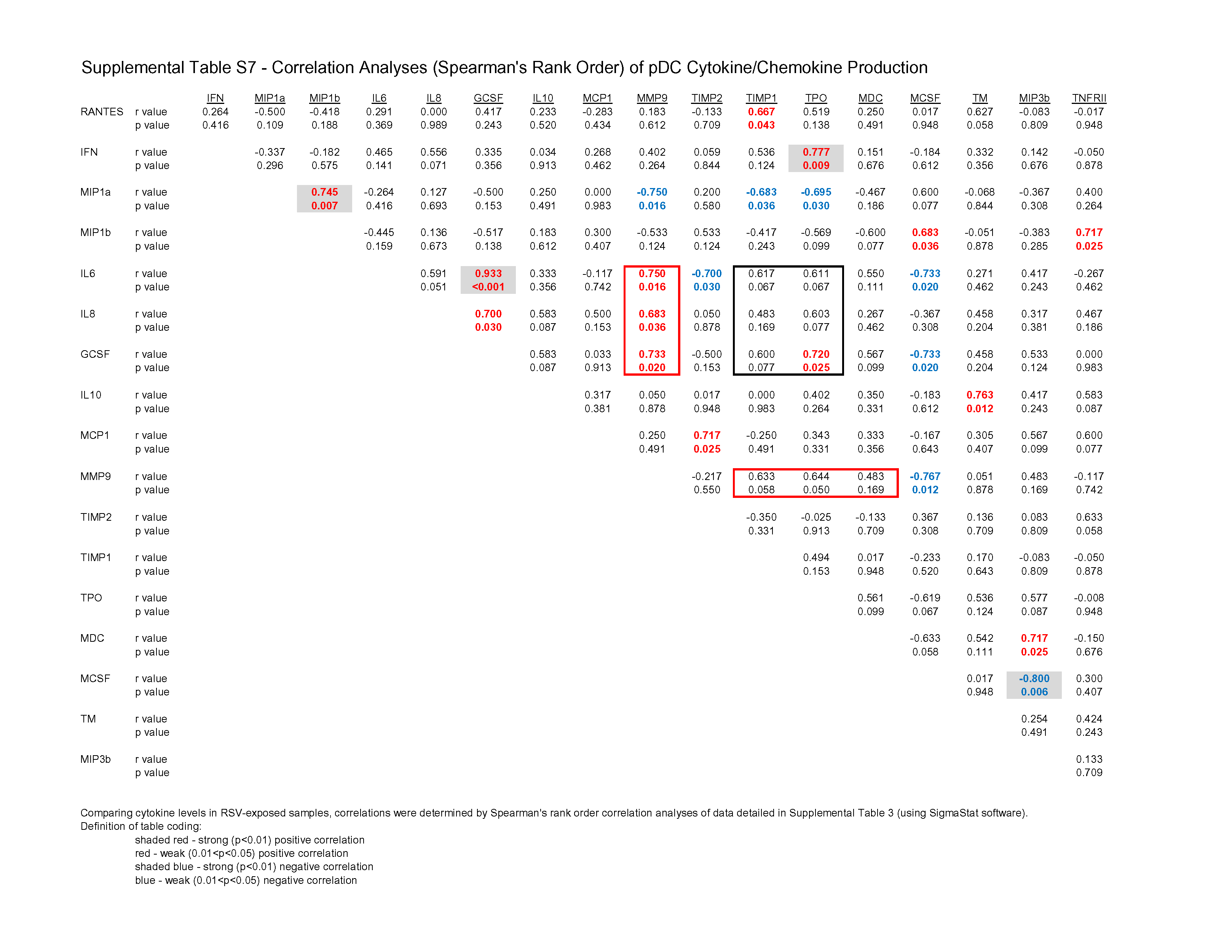

Supplement: Table S7 — Correlation Analyses (Spearman's Rank Order) of pDC Cytokine/Chemokine Production. For each individual donor (data detailed in Table S3), cytokine and chemokine production by RSV-exposed pDC were correlated to each other. These analyses were performed using Spearman's rank order test of correlation. Strong positive correlations are defined as r>0 and p<0.01 and are denoted by bold red values in gray shaded boxes while weak positive correlations are defined as r>0 and 0.01<p<0.05 and are denoted by bold red values. Similarly, strong negative correlations are defined as r<0 and p<0.01 and are denoted by bold blue values in gray shaded boxes while weak negative correlations are defined as r>0 and 0.01<p<0.05 and are denoted by bold blue values. (TIF) [file pone.0016458.s008.tif]
